# A Dengue Vaccine: Will It be Accepted and Is It Feasible? Lessons from Barranquilla, Colombia, and Merida, Venezuela

**DOI:** 10.3390/microorganisms7100458

**Published:** 2019-10-16

**Authors:** Elizabeth McMahon, Liliana Encinales, Carlos Navarro Encinales, Silvana Vielma, Nelly Pacheco, Lil Geraldine Avendaño Echavez, Sandra Acosta Rodríguez, Milena Calderon, Silvia Encinales Sanabria, Lorena Encinales Sanabria, Ericka Serrano Bernal, Andrés Gonzaléz Coba, Dennys Jiménez, Gary Simon, Aileen Y. Chang

**Affiliations:** 1 Department of Medicine, The George Washington University, Washington, DC 20037, USA; gsimon@mfa.gwu.edu (G.S.); chang@email.gwu.edu (A.Y.C.); 2 Allied Research Society, LLC, Barranquilla, Atlántico 080001, Colombia; liliana_encinales@yahoo.com (L.E.); nepamer20@hotmail.com (N.P.); 3 Fundación Hospital Universitario Metropolitano, Barranquilla, Atlántico 080001, Colombia; cana_es@hotmail.com; 4 Departament of clinical microbiology and parasitology, Universidad de los Andes, Merida, Estado Merida 5101, Venezuela; vielmasa@yahoo.com; 5 Department of Internal Medicine, Universidad Simón Bolívar, Barranquilla, Atlántico 080002, Colombia; lllil25@hotmail.com; 6 Clinica de La Costa LTDA, Barranquilla, Atlántico 080001, Colombia; spacostarodriguez@hotmail.com (S.A.R.); milecalderon@unimetro.edu.co (M.C.); sidjensa@hotmail.com (S.E.S.); lencinales@colsanjose.edu.co (L.E.S.); andresgonzalezcoba@gmail.com (A.G.C.); dennysjimenezh@hotmail.com (D.J.)

**Keywords:** dengue vaccines, feasibility studies, Colombia, venezuela

## Abstract

With one vaccine on the market and others in clinical trials, policy makers in dengue endemic regions face the decision of whether to introduce a dengue vaccine in their communities. The World Health Organization (WHO) recommends that individualized assessments be conducted before any vaccine introduction to evaluate disease burden and the strength of current vaccination programs. This study seeks to aid in that decision-making process by examining the acceptability and feasibility of dengue vaccine introduction in Barranquilla, Colombia, and Merida, Venezuela. Surveys were administered February–June of 2018 for three groups: patients (*n* = 351), health professionals (*n* = 197), and government officials (*n* = 26). In Barranquilla, most respondents reported dengue to be a moderate-severe problem, that a dengue vaccine would be useful in their communities, and that their current vaccination programs could handle the addition of a new vaccine. In Venezuela, respondents were less likely to view dengue as a major concern and listed multiple barriers to not just dengue vaccine introduction, but to providing current vaccines as well. Further work is needed in Colombia to more objectively assess the country’s readiness as a whole for a future dengue vaccine. As political and social unrest continues in Venezuela, however, future initiatives should focus on trust and capacity building. This study can serve as a framework for future assessments of the acceptability and feasibility of a dengue vaccine in both targeted areas and on larger scales.

## 1. Introduction

Dengue fever is a major public health priority throughout the Americas and much of the world. Colombia suffers from a high burden of dengue, and the primary vector, the *Aedes aegypti* mosquito, is found throughout most of the country. In the recent 2010 epidemic, 147,670 cases of nonsevere dengue, 9777 cases of severe dengue, and 217 deaths were reported in Colombia [1]. Similarly, 101,016 cases of dengue were reported in 2016, and a recent review up until epidemiological week four of 2018 already had eight reported dengue-related deaths [2]. Over 23 million individuals live in at-risk areas for dengue within Colombia [1].

Venezuela has also struggled to control dengue. From 1980–2007, Venezuela reported 35% of dengue cases as being severe, the highest rate in the Americas [3]. During the 2010 epidemic, 124,931 cases were reported. A 2010–2011 study found 77% seroprevalence of dengue in the highly endemic city of Maracay, Venezuela, with 10% of subjects showing serological markers of recent infection [4]. With the ongoing humanitarian crisis, dengue incidence in Venezuela has more than quadrupled from 1990 to 2016 [5]. As the incidence of dengue continues to grow in Colombia and Venezuela, new public health control methods are needed to limit its spread.

In 2015, Mexico registered the first dengue vaccine, Sanofi’s Dengvaxia (CYD-TDV). This prophylactic, live attenuated, tetravalent vaccine has been shown to decrease the incidence of severe dengue and hospitalization in seropositive vaccine recipients; however, it has shown to increase the above risks in vaccinees who have never been exposed to dengue prior to vaccination. In response to these recent findings, the World Health Organization (WHO) recommends prevaccination screening as to only vaccinate those who are seropositive or have a history of laboratory-confirmed dengue. Because of the increased risks and the potential for false positives with prevaccination screenings, the WHO emphasizes the importance of assessing public confidence in national vaccination programs and openness to an imperfect vaccine [6]. 

The WHO also states that countries considering introduction of a dengue vaccine should conduct careful individualized assessments. Countries must have adequate dengue surveillance systems both to gauge whether there is sufficiently high dengue seroprevalence in a region as to warrant vaccine use as well as to monitor vaccine impact and potential adverse effects after introduction [7]. Disease priority throughout the region, vaccine costs and logistics, and country capacity all need to be adequately assessed before a dengue vaccine can be successfully introduced [8]. As with any new vaccine, early preparation is key to vaccine success [9].

Colombia and Venezuela are prime candidates for introduction of a dengue vaccine, but neither has yet to implement one, and individualized assessments such as those recommended by the WHO have yet to be done. Of note, the aim of this study was to begin this process by gauging the feasibility and acceptability of dengue vaccines in a pilot location in each country, not to directly compare data between Barranquilla and Merida. Additionally, this study can serve as a framework for future individualized assessments of dengue vaccine readiness. 

## 2. Materials and Methods

The study protocol was approved by the George Washington University Institutional Review Board (IRB #091712). The study sites were located in Barranquilla, Colombia, a city of approximately 1.2 million people located along the Caribbean coast, and Merida, Venezuela, a city of approximately 205,000 in the Venezuelan Andes. Both cities are in tropical climates where dengue is endemic. 

Separate surveys were developed for each of the three subject groups of interest—patients, health professionals, and government officials. Surveys were collected from February through June of 2018. Patients were recruited from the waiting room of participating health clinics. In Colombia, these health care facilities were located in Barranquilla as well as the surrounding areas of Galapa, Baranoa, Rioviejo-Bolivar, and Soledad. In Venezuela, participating clinics included Hospital Universitario de Los Andes, Centro de Atención Médica Integral de la Universidad de Los Andes (CAMIULA, the university clinic), Unidad de Larga Eatancia, and one of the largest private clinics in the city. Providers at the same participating clinics were invited to complete the health professional survey. In Venezuela, the health professional survey was also circulated via an infectious disease WhatsApp group at the University of the Andes. Government officials were individually chosen and invited to participate in the study by local team members.

## 3. Results

### 3.1. Demographics

Between Colombia and Venezuela, survey data were gathered from 350 patients, 197 health care professionals, and 26 government officials (Table S1). Of note, 73% of patient responders in Venezuela were female. Colombian government positions included health department analysts, contractors, police central investigators, and representatives from each the National Surveillance System in Public Health (el Sistema Nacional de Vigilancia en Salud Pública), the Executive Office of the Mayor, the Secretary of Education, and human resources. Government positions represented in Venezuela included hospital and medical school directors, vaccination plan administrators, and representatives from the State Health Department as well as the offices of both the Regional Director of Epidemiology and the Director of Endemic Rural and Vector-borne Diseases.

### 3.2. Colombia

**Figure 1:**
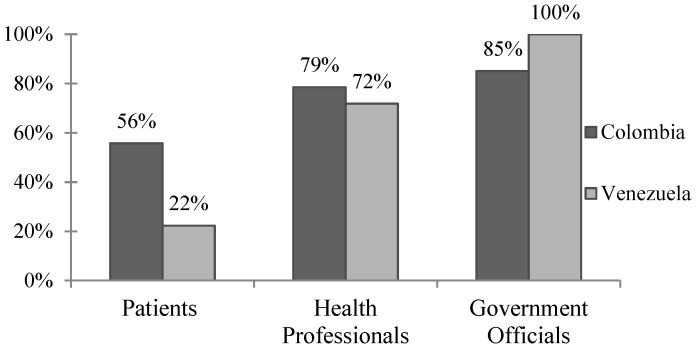
Perceived dengue severity in Colombia and Venezuela. Percentage of respondents who reported dengue is a “moderate” or “severe” problem in their communities. Responses across the three survey groups were statistically significant (*p* < 0.01) via x^2^ analysis within both Colombia and Venezuela. When contrasting results from Colombia versus Venezuela, responses were significant (*p* < 0.01) when patient and government official survey groups were compared, but not health professionals (*p* = 0.336).

The majority of patients, health professionals, and government officials rated dengue as a “moderate” or “severe” problem in their communities (Figure 1). One-third of patients reported having had dengue, and approximately one-third of those reported being hospitalized. Just under 50% of patients who reported having had dengue said they missed work or school, with an average of 8 reported days missed (Table S2). 

The vast majority of patients, health professionals, and government officials believe vaccines are beneficial in improving health and are safe (Table 1). Most also believe a dengue vaccine would be useful in their communities, and they would be willing to get themselves and their children vaccinated against dengue. Health professionals were open to a dengue vaccine requiring multiple doses, but both providers and patients were less willing to be vaccinated if protection against dengue lasts less than 2 years. Less than half of patients reported a willingness to pay for a dengue vaccine, and just 61.2% felt that it should be a standard vaccination. One-third of government officials predicted political opposition to the implementation of a dengue vaccine.

Table 2 examines the perceived logistical preparedness and capacity of Barranquilla. Notably, 94.5% of health professionals stated that their health care facility performs dengue diagnostics, with the NS1 rapid, IgM ELISA, and IgG ELISA being the most commonly reported by providers and government officials. The majority of health professionals and government officials also reported that their institutions send dengue surveillance data to a national database, that the data are electronic and collected year round, and that for reported cases of dengue there is patient follow-up. Government officials reported that data collection regarding adverse events is either active or both active and passive; however, only 70% reported that data are collected equally from public and private facilities, and that surveillance data are collected equally from all areas of the country. A total of 56.3% of government officials were “very interested” in knowing dengue serotypes in order to make public health decisions. In terms of physical capacity, 94.4% of health professionals reported that freezers and cold-chain supply are readily available; however, only 45.1% reported that their cold-chain supply has never been broken. 

**Figure 2:**
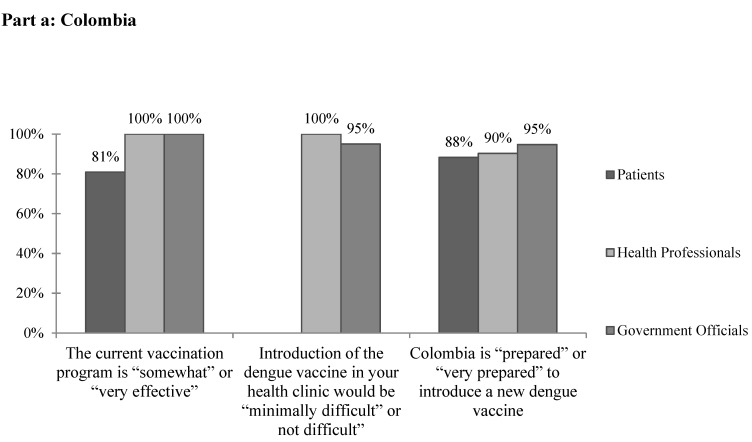

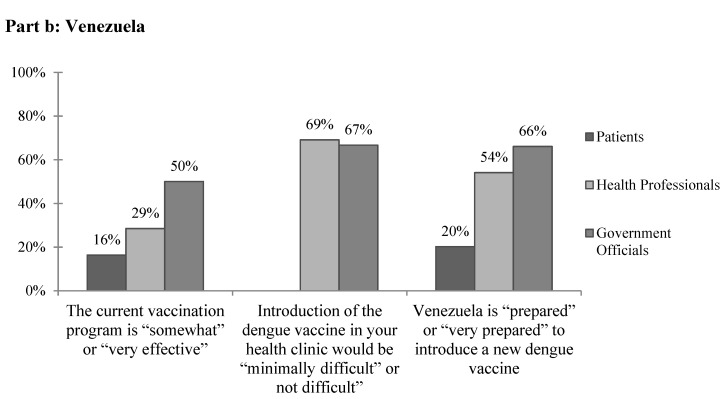
The subjective feasibility of vaccine introduction to (**a**) Colombia and (**b**) Venezuela.

A total of 92% of health professionals cited no current barriers to providing mandated vaccines (Table 3). All health professional and government official respondents and approximately 80% of patients reported that the current vaccination program is “somewhat effective” or “very effective” (Figure 2a). While 63.6% of government officials cited financial concerns when asked specifically about barriers to implementing a dengue vaccine, most health professionals selected “none.” Other barriers reported included rural areas, administrative barriers, lack of public awareness, and resistance to vaccinations. None of the government officials reported that the vaccination rate is equal in all areas of the country. On the whole, the vast majority of health professional and government respondents felt that the introduction of the dengue vaccine in their clinics would be “minimally difficult” or “not difficult.” Similarly, the vast majority of all respondents reported that Colombia is “prepared” or “very prepared” to introduce a new dengue vaccine (Figure 2a).

### 3.3. Venezuela

While most health professionals and government officials in Merida, Venezuela, reported that dengue is a “moderate” or “severe” problem in their communities, less than a quarter of patients felt the same (Figure 1). A total of 27.4% (*n* = 43) of patients reported having dengue previously, with 95.3% of them reporting confirmed cases and 18.6% reporting hospitalization. Three-quarters of patients reported missing work or school due to dengue illness for an average of 12.5 d (Table S2). 

Overall, the vast majority of respondents favored vaccines, thought a dengue vaccine would be useful in their communities, and reported a willingness to vaccinate themselves and their children (Table 4). Most respondents also reported that vaccines are safe, although government officials were less likely to say so. Despite the reported support for a dengue vaccine, only 35.8% of patients would be willing to pay for it. Few patients predicted that there would be political opposition to dengue vaccine implementation, but the majority of government officials felt there would be. Notably, a mere 1.9% of patients said information they receive from the government is true. 

With regards to capacity (Table 5), 72.6% of health professionals reported that their healthcare facility performs dengue diagnostics, with IgM ELISA the most commonly used. Only 21.3% of health professionals reported that freezers and cold-chain supply are readily available, and a mere 19.1% said that this cold chain has never been broken. One-third of health professionals and all government officials reported that vaccination plans are created each year, but few to none reported the development of multiyear plans. Similarly, 16.5% of health professionals reported that disease burden and current vaccine needs are forecasted, as did only half of government officials. In terms of surveillance logistics, only 7.4% of health professionals reported having electronic databases, as did 60% of government officials. Only 32.8% of health professionals reported that the dengue data they collect are sent to a national database, and 0.7% reported surveillance data are serotype specific. Fifty percent of government officials said that surveillance data are collected equally from all areas of the country, and 83.3% reported that data are collected equally from public and private healthcare facilities. While all government officials reported that there are current systems in place to monitor cases, lab results, vaccine coverage, adverse events, and case outcomes, one-third also stated that reported dengue surveillance data are “minimally accurate”. A total of 83.3% of government officials expressed that they are “very interested” in knowing dengue serotypes in order to make public health decisions. 

The most commonly cited barriers to providing current vaccines in Venezuela by health professionals and government officials were political (Table 6). Health professionals also frequently cited financial concerns, and government officials reported issues with vaccine availability. Other responses included unreliable electricity, lack of cold chain, patient poverty, lack of technical capacity, lack of organization, lack of foreign trade needed to buy vaccines, lack of interest, lack of supplies, logistics, and government errors. Overall, few patients and health professionals and only half of government officials reported that the current vaccination program is “somewhat” or “very effective” (Figure 2b). When asked specifically about barriers to implementing the dengue vaccine in Venezuela, both health professionals and government officials, again, most frequently cited political and financial concerns. Other reported barriers included management, maintaining cold chain, power outages, personnel, resources, transportation to far areas, maintenance of vehicles, the poor state of the current vaccination plan, barriers to vaccine entry into the country due to government policies, the epidemiologic registry, and beliefs. Of note, two-thirds of government officials reported transportation as a concern for the cost of implementing a dengue vaccine. The majority of health professionals and government officials felt that the introduction of a dengue vaccine would be “minimally difficult” or “not difficult” in their clinics, but only 20.2% of patients, 54.1% of health professionals, and 66.7% of government officials felt that Venezuela is “prepared” or “very prepared” to introduce a new dengue vaccine (Figure 2b).

## 4. Discussion

### 4.1. Overview

When considering whether to introduce a vaccine to a new area, the WHO recommends considering three factors: the disease (public health and political priority, disease burden, and other control measures), the strength of the country’s current immunization system, and characteristics of the vaccine itself [7]. This study sought to begin this process by addressing the first two factors above in the pilot cities of Barranquilla, Colombia, and Merida, Venezuela, via evaluating the attitudes of patients, health professionals, and government officials. 

### 4.2. The Disease

The WHO highlights the importance of determining whether local populations consider dengue to be a public health concern and whether or not they are open to the idea of a dengue vaccine. In the case of Barranquilla, the majority of respondents agreed that dengue is a moderate to severe problem in the community, and about a third of patients report having had dengue themselves (Figure 1, Table 2). As we currently see with the U.S. “antivaxxer” movement, public health policy makers cannot assume that all populations are open to new vaccines. Sanofi’s CYD-TDV, in particular, has received bad press, especially in the Philippines where announcement that the vaccine could be harmful to dengue-naïve individuals was highly politicized and caused a public outcry leading to a drastic drop in public confidence in not just the dengue vaccine, but vaccinations as a whole [10]. It seems, however, that these movements have not currently influenced Barranquilla, where 81–98% of all respondents agreed that vaccines are beneficial and safe, and that a dengue vaccine would be useful in their communities.

In Venezuela, there was greater disparity between survey groups, with less than a quarter of patients citing dengue as a “moderate” or “severe” problem compared with 71.8% of health professionals and all government officials (Figure 1, *p* < 0.01). This disparity may be the result of the ongoing political turmoil and health crisis in Venezuela. With rising poverty, food shortages, and unreliable electricity, it is reasonable that the general patient population may not consider dengue to be a primary concern. On the other hand, 27.4% of patient respondents self-reported having had dengue previously, and of those, just under 20% reported illness severe enough to be hospitalized. There were also discrepancies between how survey groups perceived vaccine safety, with over 85% of patients and health professionals agreeing that vaccines are safe compared to 67% of government officials. While Venezuelan respondents were less likely to agree that vaccines are safe, 88–98% felt that vaccines are beneficial in improving health and that a dengue vaccine would be useful in their communities. Thus, while the dengue vaccine seems to be accepted in Merida by patients, health professionals, and government officials, dengue illness does not appear to be a primary concern for the general population despite the recently reported dramatic increases in vector-borne diseases in the region [5].

### 4.3. Current Vaccination System

A key element in the determination of whether to introduce a new vaccine is the capacity of the region’s current vaccination system [7]. Policy makers must be able to ensure that the current vaccine delivery system is capable of delivering a dengue vaccine appropriately and can handle the added burden of a new vaccine. In Barranquilla, the vast majority of providers cited no current barriers to providing mandated vaccines. Of note, only 45% of providers reported that cold chain has never been broken in their facility, suggesting the need for further evaluation of the quality and reliability of current vaccine storage and transport methods. Overall the majority of respondents felt that the current vaccination program is “somewhat” or “very effective” (Figure 2a). However, no government officials reported that the vaccination rate is equal in all areas of the country, suggesting that these responses may differ in other, perhaps more rural, areas. While further and more objective research is required to assess the specific strengths and weaknesses of the vaccination system in place, survey respondents seem to be supportive of and confident in its current functionality.

Another important aspect to consider when evaluating a vaccination system for the introduction of a new vaccine is its surveillance capacity. This is especially important in novel vaccines such as Sanofi’s CYD-TDV and future dengue vaccines, as countries should be able to evaluate the vaccine’s effects on dengue incidence in the region as well as monitor for adverse side effects. Respondents report that the systems necessary for adequate disease surveillance and for vaccination coverage and adverse events monitoring are currently in place in Barranquilla (Table 2). Additionally, with the WHO endorsing a prevaccination strategy for CYD-TDV, it is important to examine the availability and reliability of dengue diagnostic and serologic tests. Less than 50% of government workers and less than 20% of health care providers reported using dengue IgG ELISA assays, marking a potential serious roadblock if Colombia decides to specifically adopt CYD-TDV.

Unlike in Barranquilla, in Merida there were often response discrepancies between health professionals and government officials in regards to the current vaccination system and its effectiveness. For example, only a third of health professionals reported that vaccination plans are created each year compared to all of government officials surveyed. Few health professionals reported that their databases are electronic, that multiyear vaccination plans are created, or that freezers and cold chain supply are readily available. Unlike in Colombia, only 2.2% of health professionals and none of the government officials surveyed reported no barriers to providing mandated vaccines currently, which is likely a result of Venezuela’s worsening economic and political crisis. A wide variety of complex concerns were noted including financial, political, vaccine availability, unreliable electricity, lack of technical capacity, lack of foreign trade required to purchase vaccines, and government errors. Overall, respondents reportedly lacked faith in the current vaccination system (Figure 2b).

In regards to dengue surveillance in Merida, government officials unanimously reported the existence of systems to monitor and report cases, lab results, vaccine coverage, adverse events, and case outcomes, although only about a third of providers reported that data from their facilities are sent to a national database or that follow-up exists for reported dengue cases, calling into question the validity of nationally reported data. In fact, one-third of government officials even reported current dengue surveillance data to be “minimally accurate.” While government officials reported that the framework for collecting dengue surveillance data is currently in place, further questioning revealed doubts regarding Venezuela’s current capacity to collect complete and accurate data.

### 4.4. Study Limitations

One limitation of this study is its reliance on self-reported, subjective data and the relatively small sample size (particularly in regards to government respondents). Additionally, it is important to note that Barranquilla and Merida are not representative of Colombia and Venezuela as a whole. The aim of this study, however, was to serve as a pilot in order to assess the need for larger, more comprehensive examinations of both objective and subjective measures of vaccine introduction readiness. For example, the limited interest in dengue and the lack of faith in current vaccination and surveillance programs reported in Merida suggests that such in-depth future studies are not needed in the region at this time, as further program development would be required to make vaccine introduction successful.

Another limitation of this study was the variety of positions included in the professional survey groups. For example, medical school hospital directors were included in the government officials group in Venezuela. Similarly, medical specialties represented under health professionals included personnel less likely to be involved in the direct care and management of patients with dengue including dentists, physical therapists, and occupational therapists. Thus, in future studies we would implement a specific screening process to determine under which group respondents fall as well as specialties eligible for inclusion.

## 5. Conclusions

In Barranquilla, most respondents reported that Colombia is prepared to introduce a new dengue vaccine. This is subjectively supported by respondents’ consideration of dengue as a major public health concern and their support of current vaccination systems. The next step in determining the readiness of the region for introducing a dengue vaccine would be to develop further studies that gauge more objective measures of readiness such as current vaccination rates, available budgets, localized dengue incidence, and seropositivity rates among target populations. Future work should focus on the specific population under evaluation for vaccine implementation, whether that be focal communities with high dengue prevalence, as suggested by the WHO for CYD-TDV without prescreening, or expanded to the national level for country-wide implementation. Additionally, while this study evaluated the acceptability of any dengue vaccine in general, future work should focus on the regional acceptability and logistic requirements of the specific vaccine under evaluation in the future, whether that be CYD-TDV or a novel vaccine currently in the pipeline.

In Merida, there was often incongruity between the responses of patients, health professionals, and government officials, with patients least likely to view dengue as a current problem or to support current vaccination and surveillance programs. With only 20.2% of patients and 54.1% of health professionals reporting that Venezuela is prepared to introduce a new dengue vaccine, it is clear that there is still much to be done to develop public trust and country capacity. This does not come as much of a surprise considering the ongoing humanitarian concerns and political unrest throughout the country. This study suggests that future work in Venezuela be focused on bettering its current vaccination and surveillance systems rather than the introduction of a new vaccine, which could overwhelm current programs. This study can serve as a framework for future assessments of the acceptability and feasibility of a dengue vaccine in both targeted areas and on a larger scale.

## Figures and Tables

**Table 1:** Attitudes towards vaccines. Percentage of respondents who “agree” with the following statements. NA—not applicable (the question was not posed to this survey group).

Colombia	Patients (*n* = 191)	Health Professionals (*n* = 55)	Government Officials (*n* = 20)
Vaccines are beneficial in improving health.	173 (90.6%)	54 (98.2%)	19 (95.0%)
Vaccines are safe.	156 (81.7%)	52 (94.5%)	18 (90.0%)
A dengue vaccine would be useful in your community/country.	167 (87.4%)	54 (98.2%)	19 (95.0%)
You would get a dengue vaccine.	168 (88.0%)	54 (98.2%)	18 (90.0%)
You would vaccinate your child(ren) with the dengue vaccine	156 (82.5%)	53 (96.4%)	18 (90.0%)
	*n* = 189		
You would get vaccinated if you had to receive multiple doses.	NA	48 (88.9%)	NA
You would get vaccinated if protection from illness was for less than two years.	61 (32.3%)	38 (69.1%)	NA
	*n* = 189		
Dengue vaccines should be a standard vaccination.	115 (61.2%)	50 (90.9%)	18 (90.0%)
	*n* = 188		
As a provider, you would “very likely” recommend a dengue vaccine to your patients.	NA	36 (65.5%)	NA
There will be political opposition to the dengue vaccine’s implementation.	6 (3.2%)	NA	6 (33.3%)
	*n* = 190		
You would be willing to pay for the dengue vaccine	88 (46.3%) *n* = 190	NA	NA

**Table 2:** Logistical preparedness. Percentage of respondents who answered “yes” to the following statements regarding surveillance data collection, current vaccination programs, and dengue diagnostics routinely used at their institutions. NA—not applicable (the question was not posed to this survey group). “...”—missing data.

Colombia	Health Professionals	Government Officials (*n* = 20)
Data collected are sent to a national database	52 (94.5%) *n* = 55	18 (90.0%)
Surveillance data collected are serotype specific	37 (71.3%) *n* = 52	NA
Surveillance data are collected all year round (i.e., even during the dry season)	53 (98.1%) *n* = 54	19 (95.0%)
There is currently a system in place to monitor/report:		
Cases	NA	19 (95.0%)
Lab test results	NA	20 (100%)
Vaccine coverage	NA	18 (90.0%)
Adverse events	NA	16 (88.9%) *n* = 18
Case outcomes	NA	17 (94.4%) *n* = 18
Data are collected equally from public and private healthcare facilities	NA	14 (70.0%)
Surveillance data are collected equally from all areas of the country	NA	14 (70%)
There are set national criteria in use for defining dengue (without warning signs, with warning signs, severe)	53 (100%) *n* = 53	18 (90.0%)
For reported cases of dengue there is patient follow-up	49 (94.2%) *n* = 52	18 (94.7%) *n* = 19
Databases are electronic	54 (98.2%) *n* = 55	19 (95.0%)
Vaccination plans are created each year	42 (79.2%) *n* = 53	19 (95.0%)
Multiyear vaccination plans are created	37 (72.5%) *n* = 51	13 (68.4%) *n* = 19
Disease burden/vaccine needs are forecasted	3 (6.1%) *n* = 49	9 (52.9%) *n* = 17
Freezers and cold-chain supply are readily available	51 (94.4%) *n* = 54	17 (85%)
Cold chain supply has never been broken	23 (45.1%) *n* = 51	NA
This healthcare facility performs dengue diagnostics	52 (94.5%) *n* = 54	… *n* = 16
NS1 rapid	37 (68.5%)	3 (18.8%)
IgM rapid	11 (20.4%)	6 (37.5%)
IgG rapid	8 (14.8%)	6 (37.5%)
Neutralization assay	0 (0%)	0 (0%)
IgM ELISA	12 (22.2%)	9 (56.3%)
IgG ELISA	9 (16.7%)	7 (43.8%)
NS1 ELISA	0 (0%)	0 (0%)
PCR	2 (3.7%)	4 (25.0%)

**Table 3:** Self-reported barriers to currently providing mandated vaccines and to implementing the dengue vaccine in Colombia. Free responses were then coded into the categories below.

Colombia	Health Professionals (*n* = 39)	Government Officials (*n* = 16)
Barriers to current vaccines		
Financial	1 (2.6%)	7 (43.8%)
Political	0 (0%)	0 (0%)
Vaccine Availability	0 (0%)	0 (0%)
Lack of education/dissemination of knowledge	0 (0%)	0 (0%)
Other	1 (2.6%)	5 (31.3%)
None	36 (92.3%)	5 (31.3%)
Unknown	1 (2.6%)	0 (0%)
Barriers to implementing a dengue vaccine		
Financial	6 (17.6%)	7 (63.6%)
Cultural	5 (14.7%)	4 (40.0%)
Political	0 (0.0%)	3 (27.3%)
Other	0 (0.0%)	4 (36.4%)
None	21 (61.8%)	0 (0.0%)
Unknown	3 (8.8%)	0 (0.0%)

**Table 4:** Attitudes towards vaccines. Percentage of respondents who “agree” with the following statements. NA—not applicable (the question was not posed to this survey group).

Venezuela	Patients (*n* = 159)	Health Professionals (*n* = 142)	Government Officials (*n* = 6)
Vaccines are beneficial in improving health.	151 (95.0%)	133 (93.7%)	6 (100%)
Vaccines are safe.	141 (88.7%)	121 (85.2%)	4 (66.7%)
A dengue vaccine would be useful in your community/country.	150 (95.5%)	132 (94.3%)	6 (100%)
	*n* = 157	*n* = 140	
You would get a dengue vaccine.	150 (94.3%)	132 (94.3%)	5 (83.3%)
		*n* = 140	
You would vaccinate your child(ren) with the dengue vaccine	149 (94.9%)	134 (94.4%)	6 (100%)
	*n* = 157		
You would get vaccinated if you had to receive multiple doses.	NA	130 (92.2%)	NA
		*n* = 141	
You would get vaccinated if protection from illness was for less than two years.	146 (95.4%)	125 (89.3%)	NA
	*n* = 153	*n* = 140	
Dengue vaccines should be a standard vaccination.	147 (95.5%)	127 (92.7%)	6 (100%)
	*n* = 154	*n* = 137	
As a provider, you would “very likely” recommend a dengue vaccine to your patients.	NA	118 (84.9%)	NA
		*n* = 139	
There will be political opposition to the dengue vaccine’s implementation.	24 (15.0%)	NA	4 (66.7%)
	*n* = 160		
You would be willing to pay for the dengue vaccine	57 (35.8%)	NA	NA

**Table 5:** Logistical preparedness. Percentage of respondents who answered “yes” to the following statements regarding surveillance data collection, current vaccination programs, and dengue diagnostics routinely used at their institutions. NA—not applicable (the question was not posed to this survey group). “...”—missing data.

Venezuela	Health Professionals	Government Officials (*n* = 6)
Data collected sent to a national database	44 (32.8%) *n* = 134	6 (100%)
Surveillance data collected are serotype specific	1 (0.7%) *n* = 138	NA
Surveillance data are collected all year round (i.e., even during the dry season)	91 (66.9%) *n* = 136	6 (100%)
There is currently a system in place to monitor/report:		
Cases	NA	6 (100%)
Lab test results	NA	6 (100%)
Vaccine coverage	NA	6 (100%)
Adverse events	NA	6 (100%)
Case outcomes	NA	6 (100%)
Data are collected equally from public and private healthcare facilities	NA	5 (83.3%)
Surveillance data are collected equally from all areas of the country	NA	3 (50.0%)
There are set national criteria in use for defining dengue (without warning signs, with warning signs, severe)	120 (90.2%) *n* = 133	6 (100%)
For reported cases of dengue there is patient follow-up	46 (34.3%) *n* = 134	5 (83.3%)
Databases are electronic	10 (7.4%)	3 (60.0%)
	*n* = 135	*n* = 5
Vaccination plans are created each year	46 (33.3%)	6 (100%)
	*n* = 138	
Multiyear vaccination plans are created	8 (15.4%)	0 (0%)
	*n* = 52	
Disease burden/vaccine needs are forecasted	22 (16.5%)	3 (50.0%)
	*n* = 133	
Freezers and cold-chain supply are readily available	30 (21.3%)	5 (83.3%)
	*n* = 141	
Cold chain supply has never been broken	26 (19.1%)	NA
	*n* = 136	
This healthcare facility performs dengue diagnostics	98 (72.6%) *n* = 135	...
NS1 rapid	17 (12.2%)	0 (0%)
IgM rapid	36 (25.9%)	0 (0%)
IgG rapid	25 (18.0%)	0 (0%)
Neutralization assay	0 (0%)	0 (0%)
IgM ELISA	80 (57.6%)	6 (100%)
IgG ELISA	9 (6.5%)	0 (0%)
NS1 ELISA	1 (0.7%)	0 (0%)
PCR	6 (4.3%)	0 (0%)

**Table 6:** Self-reported barriers to currently providing mandated vaccines and to implementing the dengue vaccine in Venezuela. Free responses were then coded into the categories below.

Venezuela	Health Professionals (*n* = 139)	Government Officials (*n* = 6)
Barriers to current vaccines		
Financial	60 (43.2%)	1 (16.7%)
Political	71 (51.1%)	3 (50.0%)
Vaccine Availability	16 (11.5%)	3 (50.0%)
Lack of education/dissemination of knowledge	6 (4.3%)	1 (16.7%)
Other	7 (5.0%)	2 (33.3%)
None	3 (2.2%)	0 (0%)
Unknown	3 (2.2%)	0 (0%)
Barriers to implementing a dengue vaccine		
Financial	37 (27.8%)	5 (83.3%)
Political	95 (71.4%)	3 (50.0%)
Vaccine Availability	1 (0.8%)	1 (16.7%)
Lack of education/dissemination of knowledge	6 (4.5%)	0 (0%)
Other	8 (6.0%)	2 (33.3%)
None	0 (0%)	0 (0%)
Unknown	0 (0%)	0 (0%)

## Supplementary Materials

Supplementary materials can be found at https://www.mdpi.com/2076-2607/7/10/458/s1.
