# Supplementary material for: A Dengue Vaccine: Will It be Accepted and Is It Feasible? Lessons from Barranquilla, Colombia, and Merida, Venezuela
Source: Microorganisms. 2019 Oct 16;7(10):458. doi: 10.3390/microorganisms7100458 (PMC6843364; doi:10.3390/microorganisms7100458)
Supplement: Supplementary file 1 [file microorganisms-07-00458-s001.pdf]

Table S1: Baseline demographics of patient, health professional and government official survey respondents. NA - not applicable (the question was not posed to this survey group)

**Colombia**	**Patients** (n=191)	**Health Professionals** (n=56)	**Government Officials** (n=20)
Age (mean)	39 (18-76) n=187	NA	NA
Female	93 (49.2%) n=189	34 (65%)	5 (25.0%)
Education Level			(n=18)
Did Not Finish High School	11 (5.8%)	0 (0%)	1 (5.6%)
High School	60 (31.4%)	2 (3.6%)	3 (16.7%)
Some College	24 (12.6%)	1 (1.8%)	0 (0%)
College	81 (42.4%)	41 (74.5%)	6 (33.3%)
Graduate education	17 (8.9%)	11 (20.0%)	8 (44.4%)
Position			
Nurse	NA	25 (45%)	NA
Doctor	NA	14 (27%)	NA
Other	NA	16 (29%)	NA

**Venezuela**	**Patients** (n=160)	**Health Professionals** (n=141)	**Government Officials** (n=6)
Age	41 (18-78)	NA	NA
Female	117 (73.1%)	84 (59.6%)	3 (50.0%)
Education Level	(n=159)		
Did Not Finish High School	22 (13.8%)	0 (0%)	0 (0%)
High School	57 (35.8%)	0 (0%)	0 (0%)
Some College	34 (21.4%)	0 (0%)	0 (0%)
College	39 (24.5%)	11 (8.1%)	1 (16.7%)
Graduate education	7 (4.4%)	127 (93.4%)	5 (83.3%)
Position		(n=139)	
Nurse	NA	9 (6.5%)	NA
Doctor	NA	128 (92.1%)	NA
Other	NA	2 (1.4%)	NA

Table S2: Dengue incidence/severity as self-reported by patients

	**Colombia** (n=191)	**Venezuela** (n=157)
Reported having dengue	62 (32.5%)	43 (27.4%)
Number of illness bouts (avg, range)	1.2 (1-2)	1.2 (1-3)
Reported to be lab confirmed	42 (67.7%)	41 (95.3%) n=43
Hospitalized	22 (35.4%)	8 (18.6%) n=43
Missed work/school	27 (43.5%)	51 (75.0%) n=68
Average number of days missed	8	12.5
Family member missed work/school to care for patient	12 (6.3%) n=191	36 (23.1%) n=156
Household member treated for dengue	43 (24.0%) n=179	58 (36.5%) n=159
Number of times (average, range)	1.2 (1-3)	1.6 (1-4)
